# Chronic alcoholism associated with diabetes induced apoptosis in the corpus cavernosum of rats^1^

**DOI:** 10.1590/s0102-865020200080000005

**Published:** 2020-09-07

**Authors:** Bruno Cesar Schimming, Mucio Luiz de Assis Cirino, Fermino Sanches Lizarte, Paulo Cezar Novais, Camila Albuquerque Melo de Carvalho, Daniela Pretti da Cunha Tirapelli, Carlos Augusto Fernandes Molina, Luis Fernando Tirapelli, Silvio Tucci

**Affiliations:** I PhD, Associate Professor, Department of Anatomy, Biosciences Institute of Botucatu, Universidade Estadual Paulista (UNESP), Botucatu-SP, Brazil. Technical procedures, interpretation of data, statistical analysis, manuscript preparation, critical revision.; II Fellow PhD degree, Postgraduate Program in Surgical Clinic, Department of Surgery and Anatomy, Faculdade de Medicina de Ribeirão Preto, Universidade de São Paulo (FMRP-USP), Ribeirao Preto-SP, Brazil. Acquisition and interpretation of data, statistical analysis.; III PhD, Department of Surgery and Anatomy, Surgical Clinic Program, FMRP-USP, Ribeirao Preto-SP, Brazil. Acquisition and interpretation of data, statistical analysis, critical revision.; IV PhD, Department of Odontology, Universidade de Marília (UNIMAR), Marilia-SP, Brazil. Acquisition and interpretation of data, statistical analysis.; V PhD, Department of Surgery and Anatomy, FMRP-USP, Assistant Professor, Centro Universitário Barão de Mauá, Ribeirao Preto-SP, Brazil. Acquisition and interpretation of data, statistical analysis.; VI PhD, Assistant Professor, Department of Surgery and Anatomy, FMRP-USP, Ribeirao Preto-SP, Brazil. Conception and design of the study, critical revision.; VII PhD, Assistant Professor, Department of Surgery and Anatomy, FMRP-USP, Ribeirao Preto-SP, Brazil. Conception and design of the study, critical revision.; VIII PhD, Associate Professor, Department of Surgery and Anatomy, FMRP-USP, Ribeirao Preto-SP, Brazil. Conception and design of the study, critical revision.; IX PhD, Associate Professor, Department of Surgery and Anatomy, FMRP-USP, Ribeirao Preto-SP, Brazil. Conception and design of the study, critical revision, supervised all phases.

**Keywords:** Diabetes Mellitus, Ethanol, Caspases, Rats

## Abstract

**Purpose:**

To evaluate the effects of alcohol exposure and diabetes on apoptotic process in the corpus cavernosum.

**Methods:**

Forty eight male Wistar rats were divided into four groups: control, diabetic, alcoholic and diabetic-alcoholic. Samples of the corpus cavernosum were prepared to study protein expression of apoptotic genes (Caspases-3 and 9) by immunohistochemistry and Real-Time PCR.

**Results:**

The immunoreactivity of Caspases-3 and -9 was diffuse and higher in the treated groups though there was no significant difference between the experimental groups, only when compared with the control group. An increase was observed in the gene expression of Caspases-9 in the diabetic and ethanol-diabetic groups when compared with control and ethanol groups.

**Conclusions:**

The association of these factors (ethanol and diabetes) probably can affect the apoptosis mechanism in lesions of the cavernous tissue in the rat penis. Both gene and protein expression of Caspase-9 in diabetic and ethanol-diabetic groups suggest the involvement of the apoptosis cascade from this study model.

## Introduction

Erectile dysfunction affects more than 50% of diabetic men and is approximately 3.5 times more prevalent than in non-diabetic men^1^. Among the various penile structural changes that lead to erectile dysfunction are adipocyte accumulation and apoptosis in the trabecular smooth muscle of the corpus cavernosum, for example^2^.

The association between Diabetes mellitus (DM) and erectile dysfunction has been studied for many years. Erectile dysfunction secondary to angiopathic, neuropathic and myopathic damage is one of the main complications of diabetes in men. Other reproductive complications include ejaculatory dysfunction, hypogonadism, modified semen parameters, and delayed puberty^3^. In addition to the clinical symptoms presented above, it is known that DM disrupts the functions and dynamics of mitochondria, which play a key role in regulating metabolic pathways and are crucial for maintaining proper energy balance^4^. The role of mitochondria is important in fertility. In many multisystem disorders caused by mitochondrial dysfunction, germ cell death occurs before sexual maturity^5^. Mitochondria have a variety of cellular functions and pathologies, including cell signaling, cell metabolism, cell death, aging, and cancer^6^.

Apoptosis is a genetically controlled process of programmed cell death, used by multicellular organisms to eliminate cells in diverse physiological settings, such as development, homeostasis of tissues, and maintenance of integrity of the organism^7^. The caspases are cysteine proteases and consist in the central components of the apoptotic process^8^.

The apoptosis is an event in erectile dysfunction, and pro- and anti-apoptotic factors are involved in the etiology of erectile dysfunction induced by DM. Some authors mentioned that the apoptotic index in the penis of the diabetic rat was significantly higher than that in control rat penis^9^. Same results were obtained in erectile tissues of diabetic and normal rats^10^. For these authors, the high rate of apoptosis in diabetic rats may play a role in the pathophysiology of erectile dysfunction.

Another factor that produces interference in the apoptosis process is ethanol. It has been demonstrated that high alcohol rats showed a marked thinning of the left ventricular wall combined with increased caspase-3 activity^11^. Ethanol has several effects on male sexual activity^12^. Previous studies from our group have demonstrated that chronic alcoholism induced dysfunction of smooth muscle in the corpus cavernosum of rats^13^ and decreased cavernosal smooth muscle area in rats^14^.

Although effects of ethanol on the ultrastructure of cavernous smooth muscle cells, elastic fibers and collagen content; and apoptosis in the erectile tissues of diabetic rats has been showed^10,12^, no studies have been done to analyze the apoptotic effects of the diabetes associated with ethanol in the corpus cavernosum of rats. Thus, the aim of this study was evaluate the protein expression of apoptotic genes caspase-3, and -9 in the corpus cavernosum of rats undergoing alcoholism models and induced diabetes (alloxan injection), through the techniques of Immunohistochemistry and Quantitative Real-Time Polymerase Chain Reaction (real-time PCR).

## Methods

### *Experimental design*

This study was carried out in the Molecular Biology Laboratory, Department of Surgery and Anatomy, Faculdade de Medicina de Ribeirão Preto, Universidade de São Paulo (FMRP-USP), and was approved by the Animal Use and Ethics Committee (112/2007). All experimental procedures were performed based on the Brazilian guidelines of animal welfare (11794/08).

Adult male Wistar rats (body mass 200-230g) were used. Forty eight animals were randomly divided into four groups (12 rats in each group): control (C), diabetic (D), alcoholic (A), and diabetic-alcoholic (DA). The rats in the control and diabetic groups had access to drinking water *ad libitum* and those in the alcohol and diabetic-alcohol groups received 20% (v/v) ethanol in their drinking water for three weeks. These groups underwent a brief and gradual adaptation period: the rats received 5% ethanol in their drinking water in the first week, 10% in the second week, and 20% in the third week, beginning with the pilot after the third week of treatment^15^.

Experimental diabetes mellitus was induced in rats of the diabetic and diabetic-alcoholic groups by a single i.v. injection of alloxan at the dose of 45 mg/kg into the caudal vein. It was prepared in 0.1 M/l citrate buffer (pH 4.5). The control group received citrate buffer only. All animals were bled weekly from a tail stab to measure the level of blood glucose using reagent strips (BM-ACCUTEST® and an auto-recorder, Accutrend®, Boehring Mannheim, UK). After 7 weeks, all animals were anaesthetized by i.p. injection of thiopental sodium (0.4 mg/kg) and then decapitated. The penis was collected by cutting the body of the penis at the level of its attachment to the ischium bone. The penis fragments were destined to immunohistochemistry and real-time PCR techniques.

### *Immunohistochemical examination*

The penis samples of the rats of control (n=6), diabetic (n=6), alcoholic (n=6) and diabetic-alcoholic (n=6) groups were immediately removed and fixed for 24h in ice-cold 0.1 mol/l PBS (pH 7.4), containing 4% paraformaldehyde, and destined to the histological routine with obtainment of transversal sections of the corpus cavernosum (3µm thick). Antigen retrieval was done in a microwave oven with the use of a citrate buffer solution (pH 6.0). Endogenous peroxidase was pre-blocked by using 3% hydrogen peroxide (H_2_O_2_) in methanol at room temperature. The slides were washed in PBS and incubated in 3% BSA in PBS for 1 hour at room temperature to block nonspecific staining, followed by overnight incubation in a moist chamber at 4°C with a polyclonal anti-CASPASE 3 and –CASPASE 9 antibody (Chemicon, USA) at 1:100 dilution. The slides were then washed in PBS, and a secondary antibody ((Biotinylated Rabbit Anti-Mouse Immunoglobulins - DAKO® CYT) was applied for 1 hour at room temperature, followed by three washes in PBS. The reaction was visualized by using diaminobenzidine tetrahydrochloride (DAB) solution, and sections were counterstained with Mayer’s hematoxylin. The protein expression analysis was performed in two fields (one in each corpus cavernosum) of a transversal section of penis, at 400x magnification, which had a higher concentration of positive or labeled cells (areas of “hot spots”) to related proteins of the apoptosis mechanisn (caspase 3 and caspase 9). From the count of the total number of positive cells (nuclear staining) and negative in each field, percentage of positive cells was calculated. The analysis and photodocumentation was made in light microscope Zeiss, in x400 magnification. Images were recorded by camera (Axio Cam Hrc®) attached to the microscope, filed by Axiovision 4.6® program.

### *Quantitative real-time polymerase chain reaction for caspases 3 and 9*

Total cellular RNA was extracted with Trizol Reagent (Applied Biosystems, USA) according to the manufacturer’s instructions. In preparation of Real-Time Polymerase Chain Reaction (PCR), reverse transcription of RNA samples was performed using the High-Capacity cDNA kit (Applied Biosystems, USA). For quantitative analysis of the genes Caspase-3 and -9 (Assay ID Hs00234385_m1; Rn00581212_m1, respectively), we used the commercially available system TaqMan Assay-on-demand (Applied Biosystems). The cDNA was amplified with quantitative Real Time Polymerase Chain Reaction (q-PCR) using TaqMan Master Mix (Applied Biosystems, USA) for gene reaction. The total RNA absorbed was normalized on the basis of the Ct value for β-actin (ACT-β) gene (Rn00667869-m1). All reactions were carried out in duplicate and analyzed with the 7500 Sequence Detection System apparatus (Applied Biosystems, USA). Data were analyzed using the ABI-7500 SDS software. Relative quantification of the examined genes was calculated with a calibrator by using the 2^-^method.

### *Statistical analysis*

Data were expressed as the mean value ± SEM. To evaluate the protein expression of caspases 3 and 9, statistical analysis was performed using analysis of variance tests for comparison of mean percentage among groups and multiple comparison post-test Dunnett and Bonferroni (P<0.001) was considered statistically significant. Statistical analysis of gene expression by quantitative technique of real-time PCR was analyzed by Kruskal-Wallis method, using GraphPad Prism version 4.00 for Windows (GraphPad Software, San Diego, CA, USA).

## Results

### *Immunohistochemical analyses of caspases-3 and -9*

**Figure 1:**
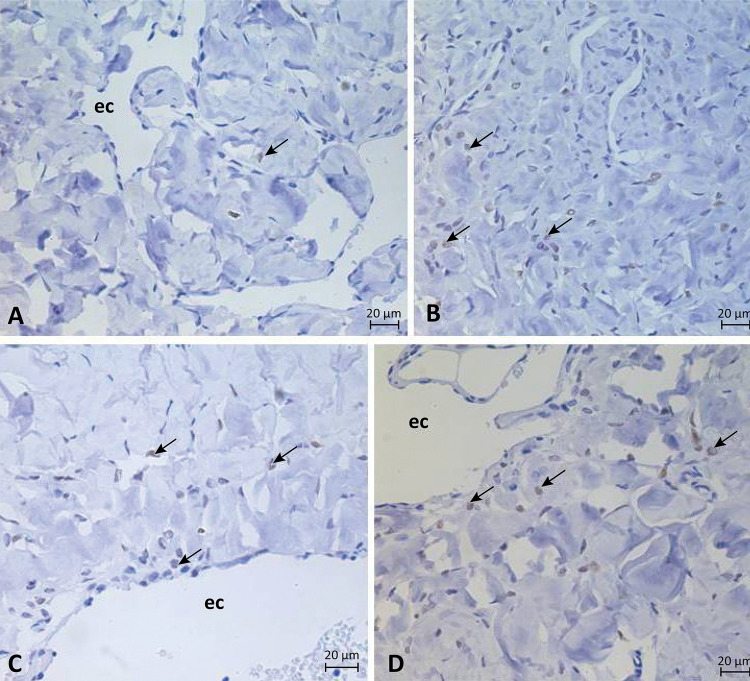
Photomicrograph showing the expression of caspase 3 in the corpus cavernosum of control (A), diabetic (B), alcoholic (C) and more diabetic-alcoholic (D) groups. Positive labeling for this protein on cell nuclei (*arrows*). Cavernous space (ec). x400

Immunohistochemical studies revealed a positive, scattered and diffuse immunoreactivity of caspase-3 in the cell nuclei throughout the cavernous tissue. This immunodetection was low in the animals of the control group. Most protein expression was found in the animals of the experimental groups, diabetic, alcoholic, and diabetic-alcoholic, when compared to the control group (Fig. 1).

**Figure 2:**
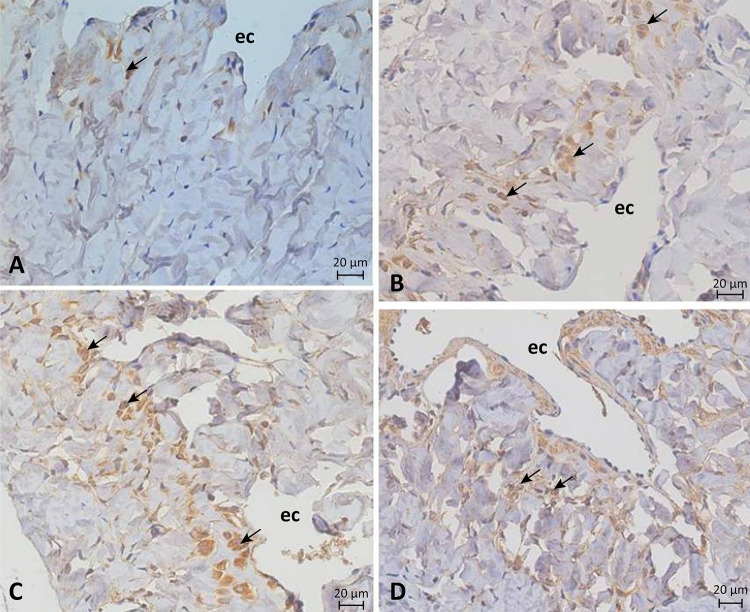
Photomicrograph showing caspase 9 expression in the corpus cavernosum of control (A), diabetic (B), alcoholic (C) and more diabetic-alcoholic (D) groups. Positive labeling for this protein on cell nuclei (*arrows*). Cavernous space (ec). x400

The caspase-9 expression in cell nuclei was dispersed and scattered around the cavernous tissue, but was higher in the endothelium lining the cavernous spaces. The immunolocalization of caspase-9 was observed weakly in the control group and sharply in animals of diabetic, alcoholic, and diabetic-alcoholic groups, when compared with the control group (Fig. 2).

### *Gene expression of caspases-3 and -9 by quantitative technique of real-time PCR*

**Figure 3:**
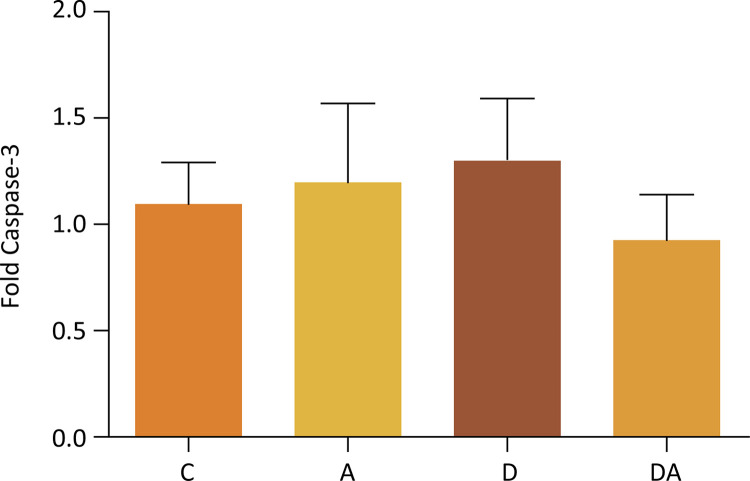
Representation of the average caspase 3 gene expression in groups C, A, D and DA (Kruskal-Willis, p=0.8063).

The gene expression of caspases-3 and -9 in the corpus cavernosum of rats in control, alcoholic, diabetic, and diabetic-alcoholic groups are showed in Figures 3 (Kruskal-Willis, p=0.8063) and 4 (Kruskal-Willis, p=0.0184), respectively. An increase in the gene expression of Caspase-9 of the diabetic and diabetic-alcohol groups was observed when compared to control and alcoholic groups.

**Figure 4:**
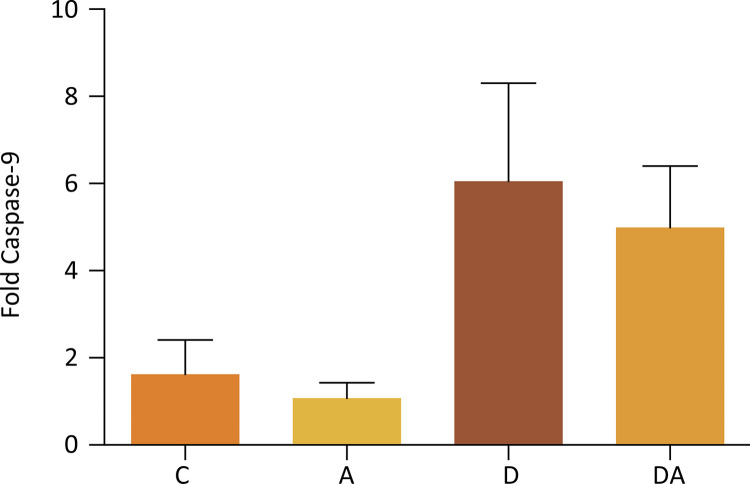
Representation of the average caspase 9 gene expression in groups C, A, D and DA (Kruskal-Willis, p=0.0184).

## Discussion

Erectile dysfunction (ED) is one of the most common disorders in males and is often associated with other comorbidities. The aging process, for example, affects the structural organization and function of erectile components of the penis, such as smooth muscle cells and vascular architecture. These changes affect penile hemodynamics, impairing the relaxation of cavernous smooth muscle cells, reducing penile elasticity, complacency and promoting fibrosis^16^. The relationship between smooth muscle cells in the corpus cavernosum and ED can be attributed to the contraction-relaxation imbalance of these cells^17^. Failures in the vascular mechanisms can lead to erectile dysfunction as well^12^. Typically, patients with diabetes mellitus develop ED early with prevalence higher than 75%. The etiology of erectile dysfunction in DM is multifactorial, characterized by peripheral neuropathy, microangiopathy and arterial insufficiency^9^.

Differentially expressed microRNAs in the corpus cavernosum of diabetic and ED rats play potential roles in the regulation of genes that are associated with the processes of apoptosis, fibrosis, vascular smooth muscle contraction, among others, mainly influencing the functions of the endothelium and smooth muscle in the corpus cavernosum^18^, although no alteration in miRNA-21 gene expression was observed in rats submitted to a chronic alcohol model^14^.

In this study, apoptosis was evaluated through protein expression of caspases-3 and -9 in the diabetic, alcoholic, and diabetic-alcoholic rats. There are no previous reports that studied the expression of these caspases with these associations. We observed that the expression of both caspases-3 and -9 increased in the experimental groups (diabetic, alcoholic, and diabetic-alcoholic), when compared with the control group. Previous studies in rats have shown that DM causes apoptosis in the corpus cavernosum. The rate of apoptotic cells in the erectile tissues of diabetic rats was significantly higher than in normal rats^9,10^. This increase of apoptosis in diabetic rats was similar to the findings of this study, where animals of diabetic and diabetic-alcoholic groups showed an increase in protein expression of caspases-3 and -9 which is indicative of apoptosis. It appears that a high rate of apoptosis in diabetic rats may have a role in the erectile dysfunction pathophysiology^10^.

The effects of DM and chronic ethanol consumption were studied by nitric oxide expression (NO) in the smooth muscle in the corpus cavernosum of rats and it was observed high expression of eNOS and iNOS in smooth muscle of diabetic rats and diabetic associated with chronic ethanol consumption when compared to control group, suggesting a decrease in the relaxation capacity induced by acetylcholine in muscles of the corpus cavernosum via endothelium-dependent mechanism, leading to suggest that chronic alcohol consumption induces endothelial dysfunction^13,19^. Ethanol consumption induces erectile dysfunction and affects the contraction and relaxation of smooth muscle of the corpus cavernosum in rats^20^.

In this study, the expression of caspase-3 was higher in alcoholic, diabetic, and diabetic-alcoholic groups when compared with control group, but there was no significant difference between the experimental groups. The increase in caspase-3 was also reported in an experimental study in rats submitted to a chronic alcohol model. These animals presented also a significant decrease in the smooth muscle area of the corpus cavernosum^14^. These authors claimed that alcoholism could affect the integrity of the smooth muscle and endothelial cells of the corpus cavernosum, and this could be important in the ED^14^.

The immunodetection of caspase-9 was also higher in the treated groups (alcoholic, diabetic, and diabetic-alcoholic) than in the control group. Regarding the caspases-3 and -9, there was no significant difference between the treated groups, only when comparing these groups with the control group. The caspase-3 immunolocalization was diffuse and not for a specific structure that comprise the cavernous tissue. It is believed that higher expression values for caspase-9 in this study in relation to caspase-3, are due to the fact that the animals are carriers of diabetes induced by alloxan and ethanol drinkers, since these values were found in diabetic and diabetic-alcoholic groups. Ethanol and DM would be responsible for an increased expression of caspase-9, which initiates apoptosis by the intrinsic pathway and would subsequently be affected by caspase-3.

The ethanol consumption associated with DM have a pivotal role in the pathogenesis of ED that is explained by reduction in the turnover of ET_A_ and ET_B_ receptors of endothelin-1 that is a potent vasoconstrictor peptide in the corpus cavernosum^21^. Thus, based on these data and on our findings, we can suggest that apoptosis should be a mechanism that cooperates to the development of erectile dysfunction associated to DM. In addition, the ethanol and the combination of ethanol and DM seem to increase apoptosis in corpus cavernosum of rats.

## Conclusions

There is an apoptosis mechanism in lesions of the cavernous tissue when subjected to alcoholism and DM models. Both gene and protein expression of caspase-9 in diabetic and diabetic-alcoholic groups suggest the involvement of the apoptosis cascade from this study model.
